# Use of schizophrenia and bipolar disorder polygenic risk scores to identify psychotic disorders

**DOI:** 10.1192/bjp.2018.89

**Published:** 2018-09

**Authors:** Maria Stella Calafato, Johan H. Thygesen, Siri Ranlund, Eirini Zartaloudi, Wiepke Cahn, Benedicto Crespo-Facorro, Álvaro Díez-Revuelta, Marta Di Forti, Genetic Risk and Outcome of Psychosis (GROUP) consortium, Mei-Hua Hall, Conrad Iyegbe, Assen Jablensky, Rene Kahn, Luba Kalaydjieva, Eugenia Kravariti, Kuang Lin, Colm McDonald, Andrew M. McIntosh, Andrew McQuillin, Psychosis Endophenotypes International Consortium (PEIC), Marco Picchioni, Dan Rujescu, Madiha Shaikh, Timothea Toulopoulou, Jim Van Os, Evangelos Vassos, Muriel Walshe, John Powell, Cathryn M. Lewis, Robin M. Murray, Elvira Bramon, Wellcome Trust Case Control Consortium 2 (WTCCC2)

**Affiliations:** 1 Division of Psychiatry, University College London, UK; 2 Division of Psychiatry, University College London, UK; 3 Division of Psychiatry, University College London, UK; 4 Division of Psychiatry, University College London and Institute of Psychiatry, Psychology and Neuroscience at King's College London and South London and Maudsley NHS Foundation Trust, UK; 5 Department of Psychiatry, Brain Centre Rudolf Magnus, University Medical Center Utrecht, the Netherlands; 6 CIBERSAM, Centro Investigación Biomédica en Red Salud Mental, Madrid and Department of Psychiatry, University Hospital Marqués de Valdecilla, School of Medicine, University of Cantabria–IDIVAL, Spain; 7 Division of Psychiatry, University College London, London, UK and Laboratory of Cognitive and Computational Neuroscience − Centre for Biomedical Technology (CTB), Complutense University and Technical University of Madrid, Spain; 8 Institute of Psychiatry, Psychology and Neuroscience at King's College London and South London and Maudsley NHS Foundation Trust, UK; 9 Psychosis Neurobiology Laboratory, Harvard Medical School, McLean Hospital, USA; 10 Institute of Psychiatry, Psychology and Neuroscience at King's College London and South London and Maudsley NHS Foundation Trust, UK; 11 Centre for Clinical Research in Neuropsychiatry, The University of Western Australia, Australia; 12 Department of Psychiatry, Brain Centre Rudolf Magnus, University Medical Center Utrecht, the Netherlands; 13 Harry Perkins Institute of Medical Research and Centre for Medical Research, The University of Western Australia, Australia; 14 Institute of Psychiatry, Psychology and Neuroscience at King's College London and South London and Maudsley NHS Foundation Trust, UK; 15 Institute of Psychiatry, Psychology and Neuroscience, King's College London and South London and Maudsley NHS Foundation Trust and Nuffield Department of Population Health, University of Oxford, UK; 16 The Centre for Neuroimaging & Cognitive Genomics (NICOG) and NCBES Galway Neuroscience Centre, National University of Ireland Galway, Ireland; 17 Division of Psychiatry, University of Edinburgh, Royal Edinburgh Hospital and Centre for Cognitive Ageing and Cognitive Epidemiology, University of Edinburgh, UK; 18 Division of Psychiatry, University College London, UK; 19 Institute of Psychiatry, Psychology and Neuroscience, King's College London and South London and Maudsley NHS Foundation Trust, UK; 20 Department of Psychiatry, Ludwig-Maximilians University of Munich and Department of Psychiatry, Psychotherapy and Psychosomatics, University of Halle Wittenberg, Germany; 21 North East London Foundation Trust and Research Department of Clinical, Educational and Health Psychology, University College London, UK; 22 Institute of Psychiatry, Psychology and Neuroscience, King's College London and South London and Maudsley NHS Foundation Trust, UK and Department of Psychology, Bilkent University, Turkey; 23 Institute of Psychiatry Psychology and Neuroscience, King's College London and South London and Maudsley NHS Foundation Trust, UK and Department of Psychiatry and Psychology, Maastricht University Medical Centre, EURON, the Netherlands; 24 Institute of Psychiatry, Psychology and Neuroscience, King's College London and South London and Maudsley NHS Foundation Trust, UK; 25 Division of Psychiatry, University College London and Institute of Psychiatry, Psychology and Neuroscience, King's College London and South London and Maudsley NHS Foundation Trust, UK; 26 Institute of Psychiatry, Psychology and Neuroscience, King's College London and South London and Maudsley NHS Foundation Trust, UK; 27 Institute of Psychiatry, Psychology and Neuroscience, King's College London and South London and Maudsley NHS Foundation Trust, UK; 28 Institute of Psychiatry, Psychology and Neuroscience, King's College London and South London and Maudsley NHS Foundation Trust, UK; 29 Division of Psychiatry and Institute of Cognitive Neuroscience, University College London and Institute of Psychiatry, Psychology and Neuroscience, King's College London and South London and Maudsley NHS Foundation Trust, UK

**Keywords:** Bipolar disorder, polygenic, prediction, psychotic disorders, polygenic risk scores, schizophrenia

## Abstract

**Background:**

There is increasing evidence for shared genetic susceptibility between schizophrenia and bipolar disorder. Although genetic variants only convey subtle increases in risk individually, their combination into a polygenic risk score constitutes a strong disease predictor.

**Aims:**

To investigate whether schizophrenia and bipolar disorder polygenic risk scores can distinguish people with broadly defined psychosis and their unaffected relatives from controls.

**Method:**

Using the latest Psychiatric Genomics Consortium data, we calculated schizophrenia and bipolar disorder polygenic risk scores for 1168 people with psychosis, 552 unaffected relatives and 1472 controls.

**Results:**

Patients with broadly defined psychosis had dramatic increases in schizophrenia and bipolar polygenic risk scores, as did their relatives, albeit to a lesser degree. However, the accuracy of predictive models was modest.

**Conclusions:**

Although polygenic risk scores are not ready for clinical use, it is hoped that as they are refined they could help towards risk reduction advice and early interventions for psychosis.

**Declaration of interest:**

R.M.M. has received honoraria for lectures from Janssen, Lundbeck, Lilly, Otsuka and Sunovian.

Psychotic disorders affect approximately 4% of the general population.^1^ Epidemiological and genetic studies show that they have high heritability.^2^^,^^3^ A Psychiatric Genomics Consortium mega-analysis of genome-wide association studies (GWAS) for schizophrenia identified more than a hundred common single nucleotide polymorphisms (SNPs) with small individual effects conferring susceptibility to the disorder.^4^ A similar mega-analysis for bipolar disorder, albeit with a more modest sample size, identified common risk variants specific to bipolar disorder and some shared with schizophrenia.^5^ Genetic epidemiology studies have shown that when compared with controls, first-degree relatives of people with schizophrenia have increased risk for bipolar disorder and first-degree relatives of people with bipolar disorder have increased risk for schizophrenia.^6^ GWAS have now provided molecular evidence for this common genetic architecture between schizophrenia and bipolar disorder.^5^^,^^7^^–^^11^ Psychotic disorders are highly polygenic with thousands of contributing common genetic variants.^12^^,^^13^ Although each individual variant has a very low predictive power, their combination into a polygenic risk score (PRS) represents a stronger predictor of disease.^8^^,^^14^^–^^20^ Our primary aim was to evaluate whether PRSs specific for schizophrenia or bipolar disorder, could discriminate case–control status in our sample of patients with broadly defined psychosis. Our secondary aim was to investigate whether PRSs were different in the unaffected relatives of patients with broadly defined psychotic disorder compared with controls.

## Method

### Sample description

Samples were collected at research centres across Europe and Australia. Our study included patients with a range of psychotic disorders (1168), unaffected relatives of patients (552) and healthy controls with no personal or family history of psychosis (1472) (Table 1). The sample presented here was included in previous GWAS seeking to identify loci for schizophrenia or psychosis. Details of sample overlap are provided in the supplement (Supplementary Data 1; available at https://doi.org/10.1192/bjp.2018.89).^4^^,^^9^^,^^21^ In order to avoid any inflation of the PRS effect size, in each analysis we included only participants that were unrelated. This was achieved by random exclusion of related participants.

**Table 1:** Demographics in the case participants, relatives and controls

	Case participants	Relatives	Controls
Age, years: mean(s.d.)	33.8 (10.2)	44.8 (15.5)	40.2 (14.3)
Gender, female: *n* (%)	386 (33)	343 (62)	763 (52)
Case participants, subdiagnosis groups, *n* (%)			
Schizophrenia	733 (62.8)		
Schizoaffective	59 (5.1)		
Bipolar disorder	109 (9.3)		
Brief psychotic disorder	43 (3.7)		
Delusional disorder	19 (1.6)		
Drug-induced psychosis	7 (0.6)		
Schizophreniform disorder	94 (8)		
Psychotic disorder not otherwise specified	104 (8.9)		
Total *n*	1168	552	1472

All participants provided written informed consent, and the study was approved by the respective ethical committees at each one of the participating centres.

Of the 1168 case participants in this study, 733 met criteria for schizophrenia (62.8%), 59 for schizoaffective disorder (5.1%), 104 for psychotic disorder not otherwise specified (8.9%), 94 for schizophreniform disorder (8%), 43 for brief psychotic disorder (3.7%), 19 for delusional disorder (1.6%), 7 for substance-induced psychosis (0.6%) and 109 for bipolar disorder with psychotic features (9.3%) (Table 1). Additional details are provided in Supplementary Tables 1 and 2 available at https://doi.org/10.1192/bjp.2018.89.

### DNA preparation, genotyping and imputation

Genomic DNA obtained from blood was sent to the Wellcome Trust Sanger Institute (Cambridge, UK). Samples were genotyped with the Genome-wide Human SNP Array 6.0 at Affymetrix Services Laboratory as part of the Wellcome Trust Case Control Consortium round 2 project (https://www.wtccc.org.uk/). Thereafter the data quality control, imputation and statistical analyses were conducted by K.L., J.T., S.C. and E.B. at University College London. DNA preparation, genotyping and imputation are described in more details in the supplement (Supplementary Data 2) and in Bramon *et al*.^9^

### Phenotype definition

Participants were excluded from the study if they had either a history of neurological disease or head injury resulting in loss of consciousness lasting more than 5 min. DSM-IV^22^ diagnosis was ascertained using a structured clinical interview with one of the following three instruments: the Schedule for Affective Disorders and Schizophrenia, the Structured Clinical Interview for DSM Disorders or the Schedules for Clinical Assessment in Neuropsychiatry.^23^^–^^25^

### Population structure analysis

To investigate the genetic structure in the data, we performed principal component analysis using EIGENSOFT version 3.0 on a pruned set of SNPs.^26^ We applied the following SNP pruning filters on 695 193 SNPs, which remained after quality control: a 10% minor allele frequency, 10^−3^ Hardy–Weinberg equilibrium deviation threshold and all SNPs within a 1500 SNP window had to have *r*^2^ below 0.2 (window shift of 150 used). Thus, a subset of 71 677 SNPs was selected for principal component analysis^26^^,^^27^ and three ancestry covariate vectors were obtained.^9^ Plots can be found in Supplementary Fig.1.

### PRSs calculation

We calculated the PRSs separately for schizophrenia and for bipolar disorder in all our study participants following established methodology.^8^^,^^28^^,^^29^ Odds ratios (ORs) of allelic association tests were obtained from the most recent Psychiatric Genomics Consortium mega-analysis of GWAS for schizophrenia^4^ and for bipolar disorder,^5^ excluding all samples overlapping with the current study. For schizophrenia, the used discovery sample included 31 658 case participants and 42 022 controls, and for bipolar disorder, it included 7481 case participants and 9250 controls.^4^^,^^5^ In each discovery samples, SNPs were selected at ten significance thresholds (*P*_T_<5 × 10^–08^, 1 × 10^−06^, 1 × 10^−04^, 1 × 10^−03^, 0.01, 0.05, 0.1, 0.2, 0.5, 1). Linkage disequilibrium pruning was used to identify SNPs in linkage equilibrium with each other. The number of SNPs included at each *P*-value threshold is shown in Supplementary Table 4. In order to obtain PRSs in each individual, for each SNP the number of risk alleles carried by the individual (0, 1, 2) was multiplied by the log of the OR of the allelic association test. The PRS was then calculated adding up the values obtained for each SNP.

### Statistical analysis

We used logistic regression, with the first three population structure principal components and the centre of ascertainment of the samples as covariates to test whether the PRSs were predictive of case–control or relative–control status in our study. The proportion of the variance explained by the PRS was calculated as Nagelkerke's pseudo-*R*^2^, by comparing a full model (PRS plus covariates) to a reference model (covariates only). The R package pROC^30^ was used to calculate the area under the receiver operator characteristic curve (AUC) in both the full and reference models.

In the primary analysis, the schizophrenia PRSs and the bipolar disorder PRSs were compared between 1168 case participants and 1472 controls. In the secondary analysis, we split the 1168 case participants with broadly defined psychosis into three subcategories, depending on the DSM diagnosis: schizophrenia/schizoaffective disorder, bipolar disorder and all other psychotic disorders. We then compared both schizophrenia and bipolar disorder PRSs between 552 unaffected relatives and healthy controls. See Supplementary Table 5 for a breakdown of these secondary analysis subgroups. In order to divide case participants and controls into decile categories, we calculated *Z*-standardised PRSs, using the mean and s.d. of controls in each centre.

## Results

### Analysis of PRSs in psychotic disorders

We calculated PRSs for schizophrenia and bipolar disorder in 1472 controls and 1168 people diagnosed with a range of psychotic disorders. Density plots of schizophrenia and bipolar disorder PRSs are shown in the Supplementary Fig. 2).

Using logistic regression, we found highly significant differences for both schizophrenia and bipolar disorder PRSs between case participants with psychosis and controls (Table 2 and Supplementary Table 6). The difference was greater for increasingly liberal *P*-value thresholds (Table 2 and Supplementary Table 6). Compared with the bipolar disorder PRSs, the schizophrenia PRSs had a better ability to discriminate between case participants and controls.

**Table 2:** Comparison of schizophrenia and bipolar disorder polygenic risk scores between patients with psychotic disorders and controls^a^

Polygenic risk score	Polygenic risk score *P*-value thresholds			
	5 × 10^−08^	1 × 10^−04^	0.05	1
Schizophrenia				
*P*-value	1.3 × 10^−06^	6.8 × 10^−21^	7.6 × 10^−40^	5.7 × 10^−40^
Variance explained, %	1.1	4.4	9	9
Bipolar disorder				
*P*-value	0.6	0.25	2.8 × 10^−09^	5.7 × 10^−11^
Variance explained, %	<0.1	<0.1	1.7	2.1

a. Schizophrenia polygenic risk scores and bipolar disorder polygenic risk scores were calculated using as reference, respectively, the outcome of the schizophrenia and bipolar disorder mega-analyses conducted by the Psychiatric Genomics Consortium. We then compared the scores between 1168 case participants and 1472 controls using standard logistic regression at ten different *P*-value thresholds (*P*_T_ 5 × 10^−08^, 1 × 10^−06^, 1 × 10^−04^, 1 × 10^−03^, 0.01, 0.05, 0.1, 0.2, 0.5, 1). Regression models included the first three ancestry-based principal components and a cohort indicator as covariates. For clarity, here we report *P*-values and the variance explained in disease risk as measured by Nagelkerke's pseudo-*R*^2^ at four *P*-value thresholds (P_T_ 5 × 10^−08^, 1 × 10^−04^, 0.05, 1). Results at each one of the ten different thresholds are available in Supplementary Table 6.

**Fig. 1:**
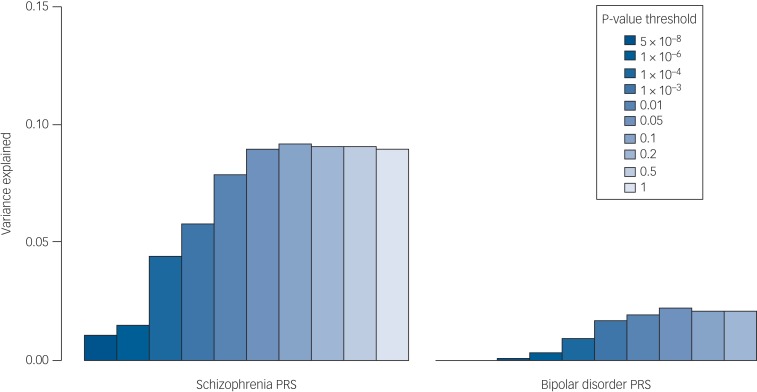
Percentage of the variance in disease risk explained by the schizophrenia and the bipolar disorder polygenic risk scores (PRSs). The proportion of variance explained (calculated as Nagelkerke's pseudo-*R*^2^) was computed by comparison of the full model (either schizophrenia-based or bipolar disorder-based PRS plus covariates) to the reduced model (covariates only). As per standard procedures,^4^ (ten different *P*-value thresholds (P_T_) were used to select risk alleles used in the computation of PRSs. The variance explained at each *P*-value threshold (5 × 10^−08^, 1 × 10^−06^, 1 × 10^−04^, 1 × 10^−03^, 0.01, 0.05, 0.1, 0.2, 0.5 and 1) is shown. Significance testing results are available in Supplementary Table S6.

The proportion of the variance in psychosis risk explained by the schizophrenia PRS increased with progressively more inclusive *P*-value thresholds, reaching a plateau of 9% variance explained at the 0.05 *P*-value threshold (Nagelkerke's pseudo-*R*^2^ = 9%; *P* = 7.6 × 10^−40^) (Table 2, Supplementary Table 6 and Fig. 1). At the same *P*-value threshold the variance explained by the bipolar disorder PRS was only 1.7% (*P*_T_ = 0.05, Nagelkerke's pseudo-*R*^2^ = 1.7%) (Table 2, Supplementary Table 6 and Fig. 1). Results for all the *P*-value thresholds used are reported in Fig. 1 and in the Supplementary Table 6.

Given that 68% of our case participants had a diagnosis of schizophrenia/schizoaffective disorder, to rule out the possibility that the results obtained were driven by this subgroup, we tested whether the schizophrenia and bipolar disorder PRSs were able to discriminate between case participants and controls in each of the three diagnostic subcategories included in our study (schizophrenia/schizoaffective disorder combined, bipolar disorder or other psychotic disorders). We demonstrated that even if the discriminative ability of the schizophrenia PRS was highest in the schizophrenia/schizoaffective disorder subcategory, it was also able to discriminate case participants with either bipolar disorder or other psychotic disorders from controls with highly significant group differences. At *P*_T_ = 0.05 the variance in case–control status explained by the schizophrenia PRS (Nagelkerke's pseudo-*R*^2^) in the bipolar disorder and other psychotic disorders subcategory was 3.4%, providing evidence that our results were not only driven by schizophrenia/schizoaffective disorder subcategory (Table 3 and Supplementary Table 7).

**Table 3:** Schizophrenia and bipolar disorder polygenic risk scores (PRSs) in the three diagnostic subgroups and in unaffected relatives *v.* controls^a^

Clinical subgroups	Schizophrenia PRS	Bipolar disorder PRS
	*P*_T_ = 0.05	*P*_T_ = 0.05
Schizophrenia/schizoaffective (*n* = 792) *v.* controls (*n* = 1472)		
*P*-value	6.1 × 10^−39^	9.2 × 10^−08^
Variance explained, %	10.3	1.6
Bipolar disorder (*n* = 109) *v.* controls (*n* = 1058)		
*P*-value	6.2 × 10^−06^	6.5 × 10^−03^
Variance explained	3.4	1.2
Other psychotic disorders (*n* = 267) *v.* controls (*n* = 1429)		
*P*-value	1.2 × 10^−08^	1.2 × 10^−03^
Variance explained, %	3.3	1.0
Relatives (*n* = 552) *v.* controls (*n* = 1221)		
*P*-value	1.2 × 10^−04^	2.1 × 10^−02^

a. Significance of the case–control PRS difference was analysed by standard logistic regression using different *P*-value thresholds (*P*_T_ 5 × 10^−08^, 1 × 10^−04^, 0.05 and 1). Here, *P*-values and Nagelkerke's *R*^2^ obtained at *P*_T_ = 0.05 are reported. Results at each one of the four different *P*-value thresholds (*P*_T_) are available in Supplementary Table 7. Logistic regression included the first three ancestry-based principal components and a cohort indicator as covariates. We report the proportion of the phenotypic variance explained by the risk polygenic score as measured by Nagelkerke's pseudo-*R*^2^.

To evaluate the accuracy of the schizophrenia and bipolar disorder PRSs in the detection of broadly defined psychotic disorders, we calculated the AUC. For the model containing only covariates (cohort and three population structure principal components) the AUC was 0.63. Adding the schizophrenia PRS to the model increased the AUC to 0.7, whereas adding the bipolar PRS increased it to 0.65 (Supplementary Fig. 3).

**Fig. 2:**
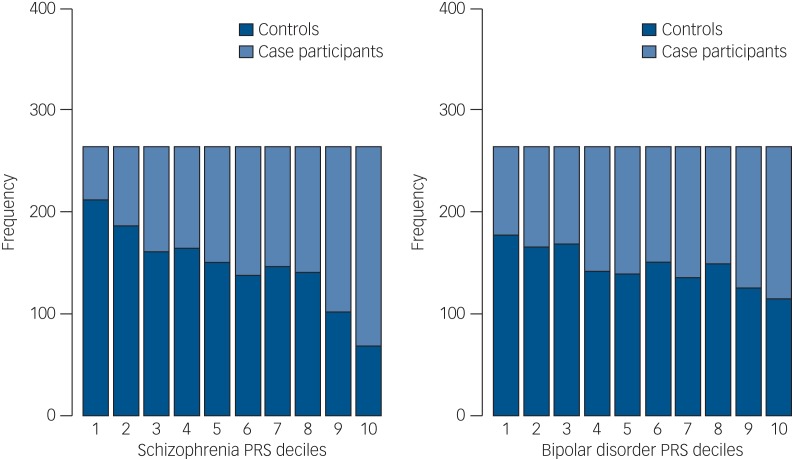
Case and control distribution in the risk polygenic score (PRS) deciles. The Y-axis corresponds to the number of individuals in each PRS decile. The *P*-value threshold used to calculate PRS was *P*_T_ = 0.05. Based on their PRS, samples were allocated to deciles (decile 1, lowest PRS; 10, highest PRS). The figure shows that especially for schizophrenia PRSs the effect is concentrated in the tails of the distribution (deciles 1–2 and 9–10). There is very little difference between the deciles 4–7 in the middle, as is expected from a normal distribution.

**Fig. 3:**
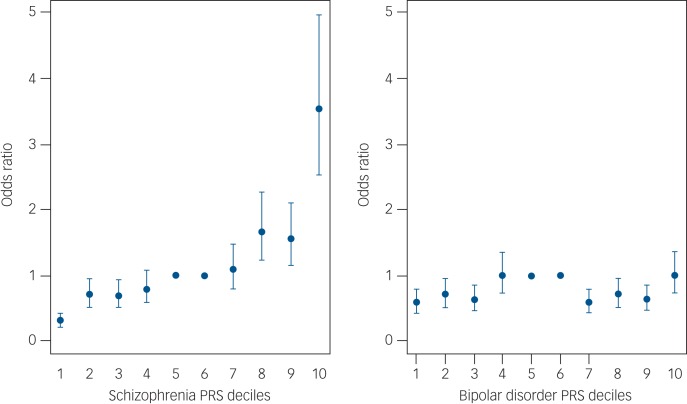
Odds ratio for broadly defined psychosis by risk polygenic score (PRS). The threshold used for selecting risk alleles was P-value threshold (*P*_T_) = 0.05. Based on PRSs, samples were allocated to deciles (decile 1, lowest PRS; 10, highest PRS). A dummy variable was created to compare the central deciles 5 and 6, used as reference to the others. Odds ratio and 95% CI were estimated using logistic regression including ethnicity principal components and cohort indicator as covariates. The points represent the odds ratios. The bars represent the lower and upper CI of the odds ratios.

We then divided our sample into deciles based on schizophrenia and bipolar disorder PRSs and calculated the ORs for affected status for each decile using as reference the central risk deciles (fifth and sixth). As expected, we observed an increase in the case-to-control ratio in progressively higher decile categories (Fig. 2 and Supplementary Table 8). Similarly, the odds of having broadly defined psychosis increased progressively across PRS deciles. Compared with individuals in the central deciles (fifth and sixth), those at the tenth and highest decile had an OR for psychosis of 3.53 (95% CI 2.53–4.97) for schizophrenia PRS (Fig. 3 and Supplementary Table 9). For the bipolar PRS no difference was found between central and highest deciles (OR = 1, 95% CI 0.73–1.35) (Fig. 3 and Supplementary Table 9).

### Analysis of PRSs in the unaffected relatives of people with psychosis

Given the established heritability of psychotic disorders, we evaluated whether schizophrenia and bipolar disorder PRSs could discriminate between unaffected relatives, who had never experienced any psychotic symptoms and healthy controls (Supplementary Fig. 4). Compared with controls, unaffected relatives had significantly higher PRSs both for schizophrenia (*P* = 1.2 × 10^−4^) and bipolar disorder (*P* = 2.1 × 10^−2^). Analyses at the *P*-value threshold of 0.05 are shown in Table 3 and full details are in Supplementary Table 7.

## Discussion

In this study, we have shown that PRSs specific for schizophrenia or for bipolar disorder obtained from a large international cohort are also associated with broadly defined psychosis in an independent sample. Compared with controls, patients with a range of psychotic disorders have significantly higher PRSs for both schizophrenia and for bipolar disorder. The schizophrenia and bipolar disorder PRSs explained, respectively, 9 and 2% of the variance in psychosis risk, which is substantial for a single variable.

The PRS for schizophrenia had a much better performance than the PRS for bipolar disorder and this could be because of several factors. First, the schizophrenia PRS contains a more accurate measure of genetic susceptibility, as it is derived from a much larger discovery sample than the bipolar PRS.^4,^^5^ The last Psychiatric Genomics Consortium schizophrenia meta-analysis provided evidence that increasing the size of discovery samples leads to a significant increase in the variance explained by PRS.^4^^,^^8^, Second, our case participants with a range of psychotic disorders included a majority of patients with schizophrenia and schizoaffective disorder (68%), which drives these performance results. However, our secondary analyses subdividing in three diagnostic categories, also showed a better performance for the schizophrenia PRS in discriminating case participants with bipolar disorder and other psychotic disorders from controls. Therefore, the use of larger discovery sample sizes appears to be the best way forward to further enhance the accuracy of PRS.^8^

GWAS have provided evidence for genetic overlap between schizophrenia and bipolar disorder.^5^^,^^7^^–^^11^^,^^14^ Our findings add evidence to the hypothesis of shared genetic architecture across the psychosis spectrum, supporting a continuum model for the aetiology of these disorders.^3^,^1^^,^^3^,^2^ The patients with bipolar disorder included in this study had type I bipolar disorder with a history of psychotic symptoms at some point in their illness. Therefore, in our sample it was not possible to make any comparison of schizophrenia and bipolar PRSs in patients with bipolar disorder with and without psychotic features. A study just published showed the existence of a gradient of schizophrenia PRSs across bipolar disorder subtypes (bipolar disorder type I with psychosis > bipolar disorder type I without psychosis > bipolar disorder type II).^3^,^3^

Given the heritability and familial aggregation patterns in schizophrenia and bipolar disorder, we expected unaffected relatives to have higher PRSs than the general population.^3^,^4^^–^^3^,^6^ In a recent study, Bigdeli *et al* showed that 217 healthy first-degree relatives of patients with schizophrenia and healthy controls could be distinguished by schizophrenia PRSs.^3^,^6^ We replicated their findings using an independent sample with 552 unaffected relatives of patients diagnosed with a wide range of psychotic disorders. Furthermore, we showed that the bipolar disorder PRS is significantly higher among healthy relatives compared with controls.

### Strengths and limitations of PRSs

Even if the schizophrenia and bipolar PRSs can discriminate case participants from controls, their accuracy is currently modest, as indicated by the AUC of 0.7 and 0.65 for schizophrenia and bipolar disorder, respectively. The AUC is an estimate of diagnostic accuracy which equals to 0.5 when a diagnostic test is no better than chance and reaches 1 if the test could discriminate patients from controls to perfection.^3^,^7^^,^^38^ Typically an AUC of 0.7 is considered to have moderate discriminatory power and only when reaching 0.9 it is deemed to have high discriminatory power.^39^^,^^40^ For example, the models used in general practice to estimate cardiovascular disease risk and to offer preventative interventions have reached AUCs in the range of 0.74–0.85.^4^,^1^^,^^4^,^2^ In the case of psychotic disorders, currently the moderate accuracy precludes the use of schizophrenia and bipolar PRSs as a diagnostic or prognostic tool in clinical practice.

Current genetic findings explain only about a third of the genetic variance of these disorders. The so-called ‘missing heritability’ may reside in further common variants yet to be identified, rare mutations, copy number variants and gene–gene interactions.^12^ As larger samples are being collected through international efforts, additional common and rare genetic variants will be identified and the performance of PRSs is expected to improve.^17^^,^^4^,^3^

In the future PRSs may also incorporate socioenvironmental factors as well as gene–gene and gene–environment interactions, thus eventually enabling their use in clinical practice for risk reduction advice as it is happening in cardiovascular disease.^4^,^4^^–^^5^,^2^ There is growing interest in the potential of PRSs in public health campaigns to reduce environmental risks and to facilitate access to early treatment for psychosis.^5^,^3^ Finally, PRSs constitute a powerful research tool, that combined with large epidemiological studies of environmental risks are advancing our understanding of the aetiology of psychotic disorders.

## Supplementary material

For supplementary material accompanying this paper visit https://doi.org/10.1192/bjp.2018.89. click here to view supplementary material
