# Supplementary material for: Use of schizophrenia and bipolar disorder polygenic risk scores to identify psychotic disorders
Source: Br J Psychiatry. 2018 Sep;213(3):535–41. doi: 10.1192/bjp.2018.89 (PMC6130805; doi:10.1192/bjp.2018.89)
Supplement: Supplementary file 1 [file S0007125018000892sup001.zip › S0007125018000892sup001/BJP_1800089_SupplementaryAppendices_TS.docx]

# Supplementary Appendix 1

## Coauthors who are members of the Psychosis Endophenotypes International Consortium (PEIC)

Maria J. Arranz^1,2^, Steven Bakker^3^, Stephan Bender^4,5^, Elvira Bramon^6,2^, Wiepke Cahn^3,^ David Collier^7,2^, Benedicto Crespo-Facorro^8,9^, Marta Di Forti^2^, Jeremy Hall^10^, Mei-Hua Hall^11^, Conrad Iyegbe^2^, Assen Jablensky^12^, René S. Kahn^3^, Luba Kalaydjieva^13^, Eugenia Kravariti^2^, Stephen M Lawrie^10^, Cathryn M. Lewis^2^, Kuang Lin^2,14^, Don H. Linszen^15^, Ignacio Mata^16,9^, Colm McDonald^17^, Andrew M McIntosh^10,18^, Robin M. Murray^2^, Roel A. Ophoff^19^, Marco Picchioni^2^, John Powell^2^, Dan Rujescu^20,21^, Timothea Toulopoulou^2,22,23^, Jim Van Os^24,2^, Muriel Walshe^6,2^, Matthias Weisbrod^25,5^, and Durk Wiersma^26^.

**PEIC affiliations:**

^1^Fundació de Docència i Recerca Mútua de Terrassa, Universitat de Barcelona, Catalonia, Spain.

^2^Institute of Psychiatry, Psychology and Neuroscience, King’s College London, De Crespigny Park, London SE5 8AF, UK

^3^University Medical Center Utrecht, Department of Psychiatry, Rudolf Magnus Institute of Neuroscience, The Netherlands.

^4^Child and Adolescent Psychiatry, University of Technology Dresden, Fetscherstrasse 74, 01307 Dresden, Germany.

^5^General Psychiatry, Vossstraße 4, 69115 Heidelberg, Germany.

^6^Division of Psychiatry & Institute of Cognitive Neuroscience, University College London, UK.

^7^Discovery Neuroscience Research, Lilly, UK.

^8^University Hospital Marqués de Valdecilla, IDIVAL, Department of Psychiatry, School of Medicine, University of Cantabria, Santander, Spain.

^9^CIBERSAM, Centro Investigación Biomédica en Red Salud Mental, Madrid, Spain.

^10^College of Biomedical and Life Sciences, Cardiff University, CF24 4HQ Cardiff, UK.

^11^Mclean Hospital, Harvard Medical School, Belmont MA, USA

^12^Centre for Clinical Research in Neuropsychiatry, The University of Western Australia, Perth, Australia

^13^Western Australian Institute for Medical Research and Centre for Medical Research, The University of Western Australia, Perth, Australia

^14^Nuffield Department of Population Health, University of Oxford, Ocford, UK

^15^Academic Medical Centre University of Amsterdam, Department of Psychiatry, Amsterdam The Netherlands

^16^Fundacion Argibide, Pamplona, Spain.

^17^The Centre for Neuroimaging &Cognitive Genomics (NICOG) and NCBES Galway Neuroscience Centre, National University of Ireland Galway, Galway Ireland

^18^Centre for Cognitive Ageing and Cognitive Epidemiology, University of Edinburgh, UK

^19^UCLA Center for Neurobehavioral Genetics, 695 Charles E. Young Drive South, Los Angeles CA 90095, USA.

^20^University of Munich, Dept. of Psychiatry, Munich, Germany

^21^University of Halle, Dept. of Psychiatry, Halle, Germany.

^22^Department of Psychology, Bilkent University, Main Campus, Bilkent, Ankara, Turkey

^23^The State Key Laboratory of Brain and Cognitive Sciences and the Department of Psychology, The University of Hong Kong, Hong Kong, China

^24^Maastricht University Medical Centre, South Limburg Mental Health Research and Teaching Network, EURON, Maastricht, The Netherlands

^25^General Psychiatry and Psychotherapy, SRH Klinikum Karlsbad-Langensteinbach, Guttmannstrasse 1, 76307 Karlsbad, Germany

^26^University Medical Center Groningen, Department of Psychiatry, University of Groningen, The Netherlands

## Coauthors who are members of the Genetic Risk and Outcome of Psychosis (GROUP) consortium

Richard Bruggeman, MD, PhD, Department of Psychiatry, University Medical Center Groningen, University of Groningen; Wiepke Cahn, MD, PhD, Department of Psychiatry, Rudolf Magnus Institute of Neuroscience, University Medical Center Utrecht; Lieuwe de Haan, MD, PhD, Department of Psychiatry, Academic Medical Center, University of Amsterdam; René S. Kahn, MD, PhD, Department of Psychiatry, Rudolf Magnus Institute of Neuroscience, University Medical Center Utrecht, Utrecht, the Netherlands; Carin Meijer, PhD, Department of Psychiatry, Academic Medical Center, University of Amsterdam; Inez Myin-Germeys, PhD, South Limburg Mental Health Research and Teaching Network, EURON, Maastricht University Medical Center; Jim van Os, MD, PhD, South Limburg Mental Health Research and Teaching Network, EURON, Maastricht University Medical Center, Maastricht, the Netherlands, and King’s College London, King’s Health Partners, Department of Psychosis Studies, Institute of Psychiatry, London, England; and Agna A. Bartels-Velthuis, PhD, Department of Psychiatry, University Medical Center Groningen, University.

# Supplementary Appendix 2

**Membership of Wellcome Trust Case Control Consortium 2 (WTCCC2)**

Management Committee: Peter Donnelly (Chair), Ines Barroso (Deputy Chair), Jenefer M Blackwell, Elvira Bramon, Matthew A Brown, Juan P Casas, Aiden Corvin, Panos Deloukas, Audrey Duncanson, Janusz Jankowski, Hugh S Markus, Christopher G Mathew, Colin NA Palmer, Robert Plomin, Anna Rautanen, Stephen J Sawcer, Richard C Trembath, Ananth C Viswanathan, Nicholas W Wood

Data and Analysis Group: Chris C A Spencer, Gavin Band, Céline Bellenguez, Colin Freeman, Garrett Hellenthal, Eleni Giannoulatou, Matti Pirinen, Richard Pearson, Amy Strange, Zhan Su, Damjan Vukcevic, Peter Donnelly

DNA, Genotyping, Data QC and Informatics Group: Cordelia Langford, Sarah E Hunt, Sarah Edkins, Rhian Gwilliam, Hannah Blackburn, Suzannah J Bumpstead, Serge Dronov, Matthew Gillman, Emma Gray, Naomi Hammond, Alagurevathi Jayakumar, Owen T McCann, Jennifer Liddle, Simon C Potter, Radhi Ravindrarajah, Michelle Ricketts, Avazeh Tashakkori-Ghanbaria, Matthew Waller, Paul Weston, Sara Widaa, Pamela Whittaker, Ines Barroso, Panos Deloukas.

Publications Committee: Christopher G Mathew (Chair), Jenefer M Blackwell, Matthew A Brown, Aiden Corvin, Mark I McCarthy, Chris C A Spencer
