# Supplementary material for: Use of schizophrenia and bipolar disorder polygenic risk scores to identify psychotic disorders
Source: Br J Psychiatry. 2018 Sep;213(3):535–41. doi: 10.1192/bjp.2018.89 (PMC6130805; doi:10.1192/bjp.2018.89)
Supplement: Supplementary file 1 [file S0007125018000892sup001.zip › S0007125018000892sup001/BJP_1800089_SupplementaryData,Figs,Tables_TS.doc]

**Supplement - Use of schizophrenia and bipolar disorder polygenic risk scores to identify psychotic disorders.**

**Supplementary Data 1**

**Sample overlap with other publications**

All the samples presented in this manuscript were also used in the Bramon et al (2014) paper. That study was aimed at identifying SNPs associated with broadly defined psychosis and included a total of 4,835 participants (1239, 857, and 2739 patients, unaffected relatives, and healthy controls respectively) ^7^. In addition, patients and controls (not the relatives) included in our study also contributed to the large international mega-analysis run by the Psychiatric Genomics Consortium ^8,9^. The aim of this large mega-analysis was the identification of new loci associated with schizophrenia.

Compared to Bramon et al 2014 and the Psychiatric Genomics Consortium studies, this current study addresses a different question: whether polygenic risk scores for both schizophrenia and bipolar disorder could be predictors of disease risk and their potential and limitations in clinical practice ^7–9^.

In order to avoid any inflation of the polygenic risk score effect size, in each analysis we included only samples that were unrelated. Therefore, patients and controls were all unrelated within and between groups; and relatives and controls were all unrelated within and between groups. This was achieved by random exclusion of related participants. Our final sample included 3,192 participants. 1,168 cases, 552 unaffected relatives, and 1,472 healthy controls (Table S1).

**Supplementary Data 2**

**DNA Sample Preparation**

Genomic DNA obtained from blood was sent to the Wellcome Trust Sanger Institute (Cambridge, United Kingdom). Samples were processed in 96-well plate format. Each plate carried a positive and a negative control. DNA concentrations were quantified using a PicoGreen assay (Invitrogen, Life Technologies, Grand Island, New York) and an aliquot assayed by agarose gel electrophoresis. A sample passed quality control if the original DNA concentration was at least 50 ng/mL and the DNA was not degraded.

**Genotyping and Quality Control**

Samples were genotyped with the Genome-wide Human SNP Array 6.0 at Affymetrix Services Laboratory ^1^ as part of the Wellcome Trust Case Control Consortium round 2 project (https://www.wtccc.org.uk/). Genotype calling was conducted at the Wellcome Trust Centre for Human Genetic (University of Oxford) using the CHIAMO algorithm modified for use with the Affymetrix 6.0 genotyping array ^2,3^. Thereafter the data quality control, imputation and statistical analyses were conducted by KL, JT, SC and EB at University College London.

We excluded 11,610 SNPs with a study-wide missing data rate over 5%. We removed 26,858 SNPs with four or more Mendelian inheritance errors identified with Pedstats ^4^. Additional exclusion criteria were departure from Hardy-Weinberg equilibrium (p < 10^–6^) or minor allele frequency (MAF) < 0.02 with 2,404 and 145,097 SNPs removed, respectively. A total of 38,895 SNPs from the X or Y chromosomes or mitochondrial DNA were also excluded from the analysis. Finally, 9,499 poorly genotyped SNPs were removed following visual inspection of the genotyping intensity plots in the program Evoker ^5^. See supplementary Table 3 for full details.

Initial analysis of the genotype data identified a high fraction of samples (approximately 30%), which showed poor signal-to-noise ratio in the genotyping assay. Because the experimental source of the problem was unclear and to ensure a robust set of genotype calls, these samples were removed from further analysis. We note that the sample loss was randomly distributed across the three clinical groups (32% of patients, 30% of relatives, and 30% of controls; χ^2^ (2 df) = 3.2; p = 0.20).

Further sample exclusion criteria were: more than 2% missing data across all SNPs, divergent genome-wide heterozygosity (inbreeding coefficients were F > 0.076 or F < −0.076 as estimated with PLINK ^6^, sex mismatch, one of each pair of individuals showing identity by descent greater than 95%.

After quality control, the full sample included 4835 participants of which 1239, 857, and 2739 were patients,

unaffected relatives, and controls, respectively ^7^.

**Phasing and Imputation**

Phasing was done using Shapeit2 v2.r790 ^10^, with default parameters except for the specification of the duoHMM flag, which allows for incorporation of known pedigree information. Imputation with reference data from the 1000 genomes panel was performed with IMPUTE2 version 2.3.0 ^11,12^. The 1000 genome reference panel was the October 2014 release, based on sequence data from 2,504 samples. Phased chromosomes where split into ~4.5 Mb chunk sizes prior to imputation and imputation was run with standard parameters assuming an effective population size of 20,000. After imputation, SNPs with poor imputation quality (INFO < 0.8) and missingness > 1% were excluded.

Table 1. Samples per research centre

| **Site** |  | **Controls** | **Cases** | **Relatives** | **Total** |
| --- | --- | --- | --- | --- | --- |
| London | Institute of Psychiatry – King’s College London | 324 | 258 | 153 | 735 |
| Edinburgh | University of Edinburgh | 21 | 31 | 0 | 52 |
| Holland | GROUP Consortium (Universities of Amsterdam, Groningen, Maastricht and Utrecht) | 713 | 378 | 298 | 1,389 |
| Australia | University of Western Australia | 162 | 301 | 92 | 555 |
| Santander | University of Cantabria | 230 | 176 | 0 | 406 |
| Heidelberg | Heidelberg University | 22 | 24 | 9 | 55 |
| **Total** |  | 1,472 | 1,168 | 552 | 3,192 |

# Table 2. Distribution of cases in sub-diagnosis groups.

|  |  | **London** | **Edinburgh** | **Holland** | **Australia** | **Santander** | **Heidelberg** | **Total** |
| --- | --- | --- | --- | --- | --- | --- | --- | --- |
| Schizophrenia/  Schizoaffective | Schizophrenia | 125 | 18 | 204 | 266 | 99 | 21 | 733 |
|  | Schizoaffective | 17 | 0 | 31 | 4 | 4 | 3 | 59 |
| Bipolar disorder | Bipolar disorder | 70 | 13 | 23 | 3 | 0 | 0 | 109 |
| Other psychotic disorder | Brief psychotic disorder | 0 | 0 | 15 | 11 | 17 | 0 | 43 |
|  | Delusional disorder | 0 | 0 | 15 | 2 | 2 | 0 | 19 |
|  | Psychosis drug induced | 2 | 0 | 5 | 0 | 0 | 0 | 7 |
|  | Schizophreniform disorder | 33 | 0 | 13 | 5 | 43 | 0 | 94 |
|  | Psychotic disorder NOS | 11 | 0 | 72 | 10 | 11 | 0 | 104 |
|  | **Total** | 258 | 31 | 378 | 301 | 176 | 24 | 1,168 |

# Table 3. Quality control filtering of SNPs.

| **Quality Control of SNPs** | **SNP Count** |
| --- | --- |
| **SNPs before quality control** | **929,556** |
| Chromosomes X, Y and mitochondrial DNA excluded SNPs | 38,895 |
| SNPs excluded due to Mendelian inheritance errors | 26,858 |
| SNPs excluded if > 5% of individuals failed genotyping | 11,610 |
| SNPs excluded if MAF <2% | 145,097 |
| SNPs excluded if HWE p value < 1 x 10^-6^ | 2,404 |
| SNPs excluded due to poor calling (manually inspected in Evoker) | 9,499 |
| **SNPs that passed all quality control filters** | **695,193** |

Hardy-Weinberg Equilibrium (HWE), minor allele frequency (MAF).

# Table 4. SNPs included in the risk polygenic score (RPS) calculation.

| **P-value Threshold** |  | **SCZ RPS** |  | **BP RPS** |
| --- | --- | --- | --- | --- |
|  |  |  |  |  |
| 5×10^-08^ |  | 70 |  | 4 |
| 1×10^-06^ |  | 166 |  | 6 |
| 1×10^-04^ |  | 963 |  | 123 |
| 1×10^-03^ |  | 2655 |  | 733 |
| 0.01 |  | 8632 |  | 4041 |
| 0.05 |  | 20515 |  | 14095 |
| 0.1 |  | 30272 |  | 23988 |
| 0.2 |  | 44237 |  | 40448 |
| 0.5 |  | 70267 |  | 77030 |
| 1 |  | 89064 |  | 108353 |

SZ RPS = schizophrenia risk polygenic score

BD RPS = bipolar disorder risk polygenic score

# Table 5. Breakdown of samples by analysis group and centre.

| **Analysis Groups** | **Site** | **Controls** | **Cases** | **Relatives** | **Total** |
| --- | --- | --- | --- | --- | --- |
|  |  |  |  |  |  |
|  | London | 324 | 258 |  | 582 |
| **Psychotic Disorders** | Edinburgh | 21 | 31 |  | 52 |
| **versus controls** | Holland | 713 | 378 |  | 1091 |
|  | Australia | 162 | 301 |  | 463 |
|  | Santander | 230 | 176 |  | 406 |
|  | Heidelberg | 22 | 24 |  | 46 |
|  | Total | 1,472 | 1,168 |  | 2,640 |
|  |  |  |  |  |  |
|  | London | 324 | 142 |  | 466 |
| Schizophrenia/Schizoaffective | Edinburgh | 21 | 18 |  | 39 |
| versus controls | Holland | 713 | 235 |  | 948 |
|  | Australia | 162 | 270 |  | 432 |
|  | Santander | 230 | 103 |  | 333 |
|  | Heidelberg | 22 | 24 |  | 46 |
|  | Total | 1,472 | 792 |  | 2,264 |
|  |  |  |  |  |  |
|  | London | 324 | 70 |  | 394 |
| Bipolar disorder | Edinburgh | 21 | 13 |  | 34 |
| versus controls | Holland | 713 | 23 |  | 736 |
|  | Australia | 0 | 0 |  | 0 |
|  | Santander | 0 | 0 |  | 0 |
|  | Heidelberg | 0 | 0 |  | 0 |
|  | Total | 1,058 | 106 |  | 1,164 |
|  |  |  |  |  |  |
|  | London | 324 | 46 |  | 370 |
| Other psychotic disorders | Edinburgh | 0 | 0 |  | 0 |
| versus controls | Holland | 713 | 120 |  | 833 |
|  | Australia | 162 | 28 |  | 190 |
|  | Santander | 230 | 73 |  | 303 |
|  | Heidelberg | 0 | 0 |  | 0 |
|  | Total | 1,429 | 267 |  | 1,696 |
|  |  |  |  |  |  |
|  | London | 324 |  | 153 | 477 |
| Relatives | Edinburgh | 0 |  | 0 | 0 |
| versus controls | Holland | 713 |  | 298 | 1011 |
|  | Australia | 162 |  | 92 | 254 |
|  | Santander | 0 |  | 0 | 0 |
|  | Heidelberg | 22 |  | 9 | 31 |
|  | Total | 1,221 |  | 552 | 1,773 |

Primary analyses were conducted on the psychotic disorder dataset versus controls. Other groups were only used for secondary analyses.

**Table 6. Schizophrenia risk polygenic score (SZ RPS) and bipolar disorder risk polygenic score (BD RPS) analysis (cases with broadly defined psychotic disorder vs controls)**

|  |  |  |  |  | **RPS P-value Threshold** | | |  |  |  |  |
| --- | --- | --- | --- | --- | --- | --- | --- | --- | --- | --- | --- |
|  |  | **5×10^-08^** | **1×10^-06^** | **1×10^-04^** | **1×10^-03^** | **0.01** | **0.05** | **0.1** | **0.2** | **0.5** | **1** |
| SZ RPS | P-value | 1.3×10^-06^ | 1.9×10^-08^ | 6.8×10^-21^ | 4.3×10^-27^ | 7.8×10^-36^ | 7.6×10^-40^ | 8.5×10^-41^ | 4.1×10^-40^ | 4.2×10^-40^ | 5.7×10^-40^ |
|  | Variance explained | 1.1% | 1.5% | 4.4% | 5.8% | 7.9% | 9% | 9.2% | 9.1% | 9.1% | 9% |
| BD RPS | P-value | 0.6 | 0.6 | 0.25 | 1.1×10^-02^ | 1.5×10^-05^ | 2.8×10^-09^ | 3.7×10^-10^ | 2.5×10^-11^ | 5.5×10^-11^ | 5.7×10^-11^ |
|  | Variance explained | <0.1% | <0.1% | <0.1% | 0.3% | 0.9% | 1.7% | 1.9% | 2.2% | 2.1% | 2.1% |

Significance of the case-control RPS difference was analysed by standard logistic regression using ten different P value thresholds (P_T_) (5×10^-08^, 1×10^-06^, 1×10^-04^, 1×10^-03^, 0.01, 0.05, 0.1, 0.2, 0.5, 1). Logistic regression included the first three ancestry based principal components (PCs) and a cohort indicator as covariates. P values and variance explained in disease risk (as measured by Nagelkerke’s pseudo R^2^) obtained the different P_T_ are reported.

**Table 7. Schizophrenia risk polygenic score (SZ RPS) and bipolar disorder risk polygenic score (BD RPS) analysis in the three diagnostic subcategories. .**

| **Diagnostic Group** |  |  | **Schizophrenia** | **RPS** |  |  |  | **Bipolar Disorder** | **RPS** |  |
| --- | --- | --- | --- | --- | --- | --- | --- | --- | --- | --- |
|  |  | **5×10^-08^** | **1×10^-04^** | **0.05** | **1** |  | **5×10^-08^** | **1×10^-04^** | **0.05** | **1** |
|  |  |  |  |  |  |  |  |  |  |  |
| Schizophrenia/Schizoaffective (n=792) | P-value | 2.8×10^-06^ | 2.7×10^-21^ | 6.2×10^-39^ | 1.1×10^-37^ |  | 0.3 | 0.8 | 9.2×10^-08^ | 1.8×10^-09^ |
| versus controls (n=1,472) | Variance explained | 1.2% | 5.1% | 10.3% | 9.9% |  | 0.1% | <0.1% | 1% | 2% |
|  |  |  |  |  |  |  |  |  |  |  |
|  |  |  |  |  |  |  |  |  |  |  |
| Bipolar disorder (n=109) | P-value | 1.1×10^-03^ | 1.3×10^-04^ | 6.2×10^-06^ | 2.9×10^-06^ |  | 0.5 | 8.4×10^-01^ | 6.5×10^-03^ | 1.2×10^-04^ |
| versus controls (n=1,058) | Variance explained | 1.7% | 2.4% | 3.4% | 3.6% |  | 0.1% | <0.1% | 1.2% | 2.4% |
|  |  |  |  |  |  |  |  |  |  |  |
|  |  |  |  |  |  |  |  |  |  |  |
| Other psychotic disorders (n=267) | P-value | 0.3 | 6.6×10^-04^ | 1.2×10^-08^ | 6×10^-09^ |  | 0.5 | 3×10^-02^ | 1.2×10^-03^ | 4.1×10^-03^ |
| versus controls (n=1,429) | Variance explained | 0.2% | 1.2% | 3.3% | 3.5% |  | <0.1% | 0.5% | 1% | 0.8% |
|  |  |  |  |  |  |  |  |  |  |  |
|  |  |  |  |  |  |  |  |  |  |  |
| Relatives (n=552) | P-value | 0.5 | 2.8×10^-03^ | 1.2×10^-04^ | 2.2×10^-06^ |  | 9.8×10^-02^ | 0.9 | 2.1×10^-02^ | 3.3×10^-02^ |
| versus controls (n=1,221) |  |  |  |  |  |  |  |  |  |  |
|  |  |  |  |  |  |  |  |  |  |  |

Significance of the case-control RPS difference was analysed by standard logistic regression using different P value thresholds (P_T_) (5×10^-08^, 1×10^-04^, 0.05, 1). Logistic regression included the first three ancestry based principal components (PCs) and a cohort indicator as covariates. P values and variance explained in disease risk (as measured by Nagelkerke’s pseudo R^2^) obtained at four different P_T_ (5×10^-08^, 1×10^-04^, 0.05, 1) are reported.

Table 8. Case to control ratios across the Risk Polygenic Score deciles

|  |  | **Schizophrenia** | | **RPS** |  |  |  | **Bipolar Disorder** | | **RPS** |  |
| --- | --- | --- | --- | --- | --- | --- | --- | --- | --- | --- | --- |
| **Decile** | **Controls** | **%** | **Cases** | **%** | **Case/Control ratio** |  | **Controls** | **%** | **Cases** | **%** | **Case/Control ratio** |
| **1** | 212 | 80.3 | 52 | 19.7 | 0.25 |  | 178 | 67.42 | 86 | 32.58 | 0.48 |
| **2** | 187 | 70.83 | 77 | 29.17 | 0.41 |  | 166 | 62.88 | 98 | 37.12 | 0.59 |
| **3** | 162 | 61.36 | 102 | 38.64 | 0.63 |  | 169 | 64.02 | 95 | 35.98 | 0.56 |
| **4** | 164 | 62.12 | 100 | 37.88 | 0.61 |  | 142 | 53.79 | 122 | 46.21 | 0.86 |
| **5** | 151 | 57.2 | 113 | 42.8 | 0.75 |  | 139 | 52.65 | 125 | 47.35 | 0.9 |
| **6** | 138 | 52.27 | 126 | 47.73 | 0.91 |  | 151 | 57.2 | 113 | 42.8 | 0.75 |
| **7** | 147 | 55.68 | 117 | 44.32 | 0.8 |  | 136 | 51.52 | 128 | 48.48 | 0.94 |
| **8** | 140 | 53.03 | 124 | 46.97 | 0.89 |  | 150 | 56.82 | 114 | 43.18 | 0.76 |
| **9** | 102 | 38.64 | 162 | 61.36 | 1.59 |  | 126 | 47.73 | 138 | 52.27 | 1.1 |
| **10** | 69 | 26.14 | 195 | 73.86 | 2.83 |  | 115 | 43.56 | 149 | 56.44 | 1.3 |

The threshold used to calculate Risk Polygenic Scores (RPS) was PT = 0.05. Based on their RPS samples were divided into deciles (decile 1 = lowest RPS, 10 = highest RPS)..

RPS = risk profile score. The table reports the case to control ratios for broadly defined psychosis across the deciles.

**Table 9. Risk polygenic score (RPS) deciles and odd ratios (OR) of broadly defined psychotic disorder**

|  | **Schizophrenia** | **RPS** |  |  | **Bipolar Disorder** | **RPS** |  |
| --- | --- | --- | --- | --- | --- | --- | --- |
| **Decile** | **OR** | **LCI** | **UCI** |  | **OR** | **LCI** | **UCI** |
| **1** | 0.30 | 0.21 | 0.43 |  | 0.58 | 0.42 | 0.80 |
| **2** | 0.70 | 0.51 | 0.95 |  | 0.70 | 0.51 | 0.95 |
| **3** | 0.69 | 0.50 | 0.94 |  | 0.63 | 0.46 | 0.86 |
| **4** | 0.79 | 0.58 | 1.07 |  | 1.00 | 0.73 | 1.35 |
| **5 and 6** | 1.00 |  |  |  | 1.00 |  |  |
| **7** | 1.09 | 0.80 | 1.48 |  | 0.58 | 0.42 | 0.80 |
| **8** | 1.66 | 1.23 | 2.26 |  | 0.70 | 0.51 | 0.95 |
| **9** | 1.55 | 1.15 | 2.11 |  | 0.63 | 0.46 | 0.86 |
| **10** | 3.53 | 2.53 | 4.97 |  | 1.00 | 0.73 | 1.35 |

Data are odds ratios (OR) and 95% confidence interval (CI). OR were calculated using the central deciles (5^th^ and 6^th^) as reference group. LCI = lower confidence interval. UCI = upper confidence interval. RPS = risk profile score

**A**

**B**

Figure 1. Ancestry principal components analysis. The figure shows a projection of the 3,192 study individuals for (A) to the first two, and (B) the first three principal components. Samples are coloured according to recruitment locations (cohort) to show their ancestry backgrounds. Ancestry principal components: PC1 = first principal component, PC2 = second principal component, PC3 = third principal component.

# Figure 2. Distribution of schizophrenia risk profile score and bipolar disorder risk polygenic score (RPS). Controls (light blue) and patients with broadly defined psychotic disorder (pink). Both schizophrenia and bipolar disorder risk profile scores are standardised scores calculated using the P-Value threshold of 0.05. RPS = risk profile score

# Figure 3. Area under the curve of the receiver operator characteristic curves (AUC) for the Schizophrenia and Bipolar Disorder risk polygenic score (RPS). SCZ_RPS = model including Schizophrenia Risk Polygenic Score and covariates. BD_RPS = model including Bipolar Disorder Risk Polygenic Score and covariates

# Figure 4. Box plots of the schizophrenia and bipolar disorder risk polygenic score (RPS) in patients with broadly defined psychotic disorder, unaffected relatives and controls. Both SZ and BD risk polygenic scores are standardised scores calculated using the P-Value threshold of 0.05.

**Supplementary references**

1	Affymetrix. Affymetrix. 2015.

2	Marchini J, Howie B, Myers S, McVean G, Donnelly P. A new multipoint method for genome-wide association studies by imputation of genotypes. *Nat Genet* 2007; **39**: 906–13.

3	Wellcome T, Case T, Consortium C. Genome-wide association study of 14 , 000 cases of seven common diseases and. *Nature* 2007; **447**: 661–78.

4	Wigginton JE, Abecasis GR. PEDSTATS: Descriptive statistics, graphics and quality assessment for gene mapping data. *Bioinformatics* 2005; **21**: 3445–7.

5	Morris JA, Randall JC, Maller JB, Barrett JC. Evoker: A visualization tool for genotype intensity data. *Bioinformatics* 2010; **26**: 1786–7.

6	Purcell S, Neale B, Todd-Brown K, Thomas L, Ferreira MAR, Bender D, *et al.* PLINK: A Tool Set for Whole-Genome Association and Population-Based Linkage Analyses. *Am J Hum Genet* 2007; **81**: 559–75.

7	Bramon E, Pirinen M, Strange A, Lin K, Freeman C, Bellenguez C, *et al.* A genome-wide association analysis of a broad psychosis phenotype identifies three loci for further investigation. *Biol Psychiatry* 2014; **75**: 386–97.

8	Ripke S, O’Dushlaine C, Chambert K, Moran JL, Kähler AK, Akterin S, *et al.* Genome-wide association analysis identifies 13 new risk loci for schizophrenia. *Nat Genet* 2013; **45**: 1150–9.

9	Ripke S, Neale BM, Corvin A, Walter JTR, Schizophrenia Working Group of the Psychiatric Genomics Consortium. Biological insights from 108 schizophrenia-associated genetic loci. *Nature* 2014; **511**: 421–7.

10	Delaneau O, Zagury J-F, Marchini J. Improved whole-chromosome phasing for disease and population genetic studies. *Nat Methods* 2013; **10**: 5–6.

11	Howie B, Fuchsberger C, Stephens M, Marchini J, Abecasis GR. Fast and accurate genotype imputation in genome-wide association studies through pre-phasing. *Nat Genet* 2012; **44**: 955–9.

12	Howie B, Marchini J, Stephens M, Chakravarti a. Genotype Imputation with Thousands of Genomes. *G3&#58; Genes|Genomes|Genetics* 2011; **1**: 457–70.
